# Classification of Depression Through Resting-State Electroencephalogram as a Novel Practice in Psychiatry: Review

**DOI:** 10.2196/19548

**Published:** 2020-11-03

**Authors:** Milena Čukić, Victoria López, Juan Pavón

**Affiliations:** 1 HealthInc 3EGA Amsterdam Health and Technology Institute Amsterdam Netherlands; 2 Instituto de Tecnología del Conocimiento, Institute of Knowledge Technology Universidad Complutense Madrid Ciudad Universitaria s/n, 28040 Madrid Spain

**Keywords:** computational psychiatry, physiological complexity, machine learning, theory-driven approach, resting-state EEG, personalized medicine, computational neuroscience, unwarranted optimism

## Abstract

**Background:**

Machine learning applications in health care have increased considerably in the recent past, and this review focuses on an important application in psychiatry related to the detection of depression. Since the advent of computational psychiatry, research based on functional magnetic resonance imaging has yielded remarkable results, but these tools tend to be too expensive for everyday clinical use.

**Objective:**

This review focuses on an affordable data-driven approach based on electroencephalographic recordings. Web-based applications via public or private cloud-based platforms would be a logical next step. We aim to compare several different approaches to the detection of depression from electroencephalographic recordings using various features and machine learning models.

**Methods:**

To detect depression, we reviewed published detection studies based on resting-state electroencephalogram with final machine learning, and to predict therapy outcomes, we reviewed a set of interventional studies using some form of stimulation in their methodology.

**Results:**

We reviewed 14 detection studies and 12 interventional studies published between 2008 and 2019. As direct comparison was not possible due to the large diversity of theoretical approaches and methods used, we compared them based on the steps in analysis and accuracies yielded. In addition, we compared possible drawbacks in terms of sample size, feature extraction, feature selection, classification, internal and external validation, and possible unwarranted optimism and reproducibility. In addition, we suggested desirable practices to avoid misinterpretation of results and optimism.

**Conclusions:**

This review shows the need for larger data sets and more systematic procedures to improve the use of the solution for clinical diagnostics. Therefore, regulation of the pipeline and standard requirements for methodology used should become mandatory to increase the reliability and accuracy of the complete methodology for it to be translated to modern psychiatry.

## Introduction

As the World Health Organization has warned since 2007, depression may become the most frequent cause of global disability by 2030 [1]. Only 11% to 30% of all patients diagnosed with depression reach remission within their first year of treatment [2,3]. It is possible that an individual may be diagnosed with more than one disorder (or 2 individuals showing completely different symptoms may be labeled with the same disorder), according to the current Diagnostic and Statistical Manual of Mental Disorders, fourth edition (DSM-IV) manual. Unlike many other medical specializations, psychiatry does not use objective physiological tests in its diagnostic process [4]. Many clinicians, health care providers, and researchers are aware that this diagnostic process needs improvement. Matching patients with interventions, finding specific biomarkers, and identifying various technical solutions can provide the much-needed improvement in clinical care.

Combining the knowledge and methodology used in computational neuroscience and psychiatry results in a discipline known as computational psychiatry. This field aims to determine the neurobiological underpinnings behind clusters of clinical symptoms, making it easier to adjust the treatment to patients on an individual level [5-7]. Among other studies applying this combination of approaches to resolve current issues with psychiatric diagnostics, Tokuda et al [8] offered impressive findings in their work in 2018. They combined demographic data, magnetic resonance imaging (MRI), and previous medical information on patients with applied statistical learning approaches to differentiate the 3 subtypes of depression.

Computational psychiatry may be divided into 2 approaches: theory driven and data driven. The data-driven approach typically involves some type of machine learning and appears to be much more applicable than the theory-driven approach owing to the comparably lower data collection costs. Although the most popular work published over the last period applies the data-driven approach through the use of MRI or functional MRI (fMRI) data, the drawbacks of this approach are the subject of debate among researchers. In our opinion, it would be much more appropriate to rely on electroencephalographic data, given the lower costs and higher patient accessibility. Electroencephalogram (EEG) is the oldest form of neuroimaging (1924, Hans Berger) and is noninvasive and solidly based on neurology and neuroscience. In psychiatry, it is only used to confirm the existence of epileptiform. As compared with fMRI, for example, EEG is more suitable for frequent testing owing to the lesser time required for recording and the lower price of processing. Witten and Frank [9] described data mining as “the extraction of implicit, previously unknown, and potentially useful information from the data,” and at present, popular machine learning forms a part of that discipline. A typical pipeline in this framework includes recording the EEG, managing artifact removal (manually, using software, or using artifact-free epochs), linear or nonlinear electroencephalographic analysis, feature extraction, feature selection, and the application of the machine learning (both training and testing phases) method of choice. As it contains highly structured data, the EEG (a matrix of voltage values as columns/recorded from different electrode voltage and time) is highly suitable for machine learning [10].

Another research area, physiological complexity, continues to be considered novel by many medical professionals. It is based on a complex systems dynamic theory (commonly called the *chaos theory*) and is made up of vast families of distinct analysis approaches in a mathematical sense. Many researchers currently use these methods given that physiological signals are known to be nonlinear and nonstationary and generated from a highly complex system that tends to operate far from the equilibrium state. The application of a mechanistic approach (suitable for stationary signals) for the analysis of electrophysiological data, which are nonlinear, nonstationary, and noisy (3N), runs the risk of flawed interpretation. Recently, research has suggested that a mathematical link exists between the commonly applied Fourier analysis and fractal analysis [11], and the use of Fourier before the latter seems to be redundant. Klonowski [12] showed that the omnipresent classical spectral analysis in electrophysiology is based on its deeply rooted use in medicine; nonlinear analysis tends to be applied in research areas only. For a review of the varying nonlinear methodologies in detecting depression based on EEG, refer to the study by de la Torre-Luque and Bornas [13,14].

Over the past 10 years, the number of research studies using some form of machine learning on an EEG data set to detect depression or predict treatment outcomes related to the same is booming. This study aims to review the literature to offer a cross-section that is useful for determining current best practices. We have chosen to focus on the combination of physiological complexity (the application of nonlinear measures of analysis of EEG) and data-driven computational psychiatry approaches, as we believe that this combination may offer faster improvement in current clinical practices focusing on the treatment of depression.

## Methods

This systematic literature review aims to find and compare published studies using nonlinear (and spectral) methods of analysis in combination with various machine learning methods for the detection of depression. Therefore, we established an inclusion criteria, as we were aware that many studies were published over the past decade. As we followed the literature for a significant amount of time, we established a start date of 2008 and an end date of May 2019.

### Search Method

Given the rapid development in this research area because of faster computers, cloud utilization, and improved internet performance, we believe that this is a sufficient inclusion period. We systematically searched the Web of Science and PubMed databases on May 24, 2019, using the following combination of keywords: (“Data mining” OR “machine learning”) AND (“EEG” OR “Electroencephalography”) AND (“Depression” OR “MDD”).

In addition, databases indexing both fields, such as Springer, Scopus, and ScienceDirect, were searched for relevant literature, including the Cornell repository.

After an original search yielding 197 papers, we reviewed all the titles and abstracts to determine which were in line with our search criteria.

### Inclusion Criteria

Our eligibility criteria (eligibility testing) consisted of the following requirements: a study published between 2008 and 2019, detection of depression or predicting the outcome of treatment for depression, sample consisting of patients diagnosed with depression (major depressive disorder [MDD]) and healthy controls (HCs), EEG data set (preferably resting-state EEG), use of fractal and nonlinear analysis as features for machine learning, and use of machine learning for detection of depression. After a primary selection phase (in which we read all the publications independently), our sample consisted of 32 publications, which was decreased to 26 based on internal discussion and comparative analysis. After reading the entire text of each publication, we decided to include 14 detection studies and 12 interventional studies. In short, we only included EEG studies that were published over the past 12 years, using task classification performed by humans with electroencephalographic signals (excluding power analyses only, nonhuman feature selection, or those with no end classification studies) that carried out a machine-based learning task aimed at detecting depression. Many studies described mobile phone apps and web-based data collection (web-based psychiatry) using machine learning, but this has already been reviewed in another work [15].

### Comparisons Considered

Before conducting this systematic search, we created a list of study characteristics for comparison and to discuss the best practices and results. First, we compared the sample sizes, with only 1 intervention study being sufficiently large to analyze a sample of over 100 participants (and only 1 study consisted of only female subjects [16]). As we chose to only include EEG studies, we divided these based on resting-state EEG (employed in diagnostics) and those using any type of stimulus during the recording.

Our idea, from a nonlinear analysis perspective, is useful for analyzing resting-state records, as previous research has shown that they are the most information-rich [17]. Berman et al [18] showed that in depression, ruminative activities may only be detected in task-free and resting-state EEG recordings. Studies also varied in the number of electrodes used for recording as well as the standards used.

The next stage of comparison considered the method used for data preprocessing, some of which used standard subbands (although there is yet to be any published data or evidence that dividing EEG into subbands has any physiological significance [19]) and others used the broadband signal. Some used reductionist approaches (such as Fourier analysis or wavelet or cosine transform) and others analyzed the raw signal. Some removed the artifact manually (probably introducing other sources of artifacts in that way), removed the artifact (automatically) with some software, or chose to analyze the epochs from artifact-free sections of recorded signal (ie, no artifact removal). Another point of discussion referred to the extent to which filtering and preprocessing were performed and whether researchers focused on any specific part of the signal’s spectral content. We also compared the sampling frequency that was applied to the raw signals, an important factor for the interpretation of results. The next stage considered the type of analysis performed on previously preprocessed data and the chosen features for further machine learning.

Studies also differed in how they chose to extract or select the features.

We also noted whether internal and external cross-validation was performed (and reported) and whether the study could potentially be replicated. Finally, we compared the methods of machine learning used in each work as well as their accuracy after the testing phase and their sensitivity and specificity. Another question considered was whether the studies used receiver operating characteristic (ROC) curves to verify their accuracy. We attempted to carry out an exhaustive analysis of those publications that complied with our eligibility criteria.

## Results

### Diagnostic Studies

**Figure 1:**
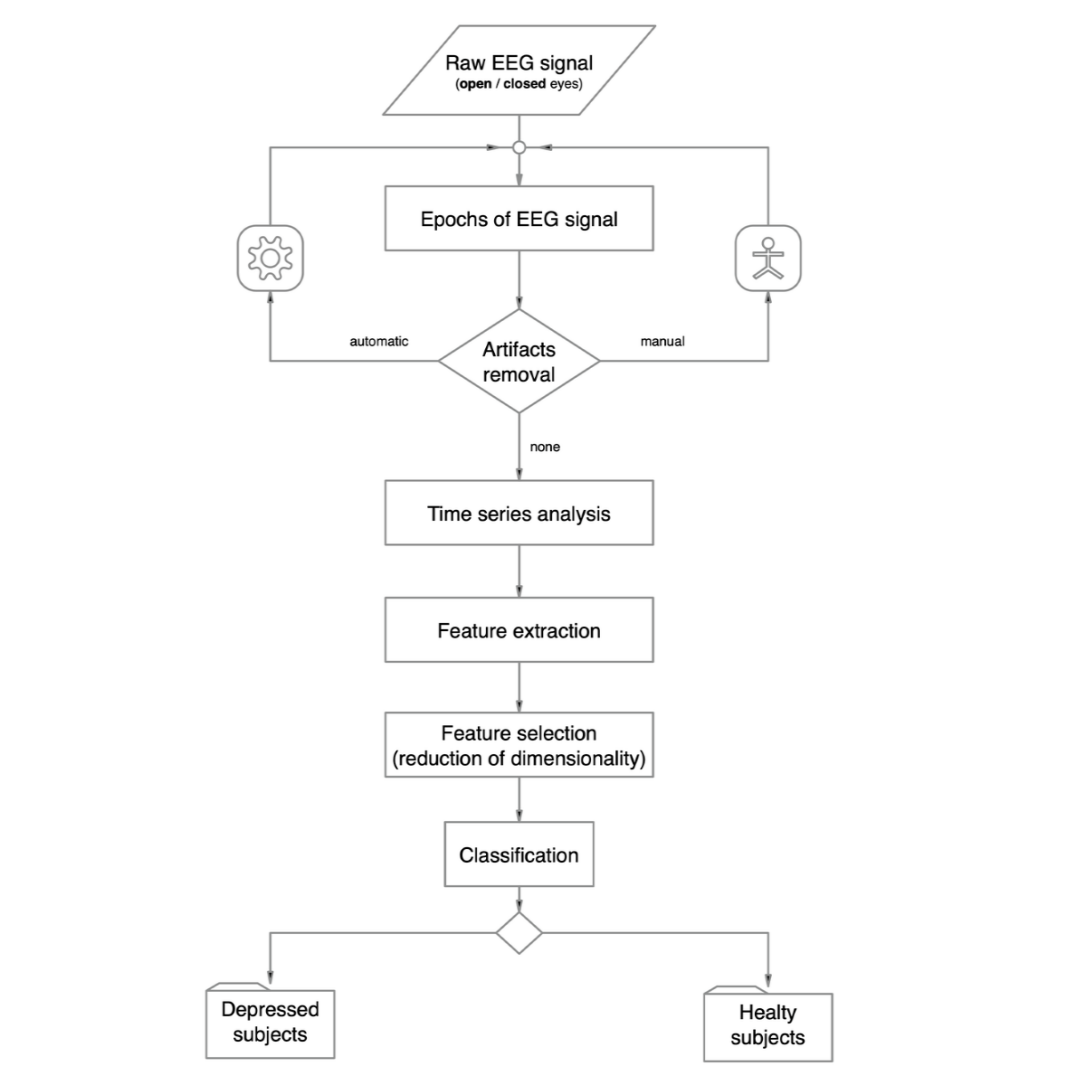
Flow diagram showing common stages in the analysis of resting-state EEG in all studies with varying approaches to classification. EEG: electroencephalogram.

We reviewed 14 studies (classified as *detection studies*) published between 2008 and 2019. The problem with this cohort of studies is similar to that of studies trying to elucidate changes in complexity from the EEG of a patient who has depression; making a direct comparison is challenging because of the distinct methodologies employed. As we wished to draw conclusions regarding the possible practical significance of these studies and found their methodologies to be quite different in many technical aspects, we compared several basic characteristics that could potentially affect their outcomes [20]. In general, the combination of the choice of features and classification model is considered to be the most important. Nevertheless, all the studies discussed here used common processing stages, as illustrated in Figure 1.

After recording the EEG (based on the previously specified method on resting-state EEG recorded with open or closed eyes, the method used to confirm the depression status, whether patients were medicated, what EEG recording standard was used, and how many electrode positions were involved), the preprocessing phase followed. Apart from standard filtering and the selection of sampling frequency, in physiological terms, the most important part involved artifact removal (manual, automatic, or no removal at all). After defining exact epochs for analysis (or better time series for further analysis), the following steps were discussed: feature extraction, feature selection (or dimensionality reduction phase), classification, validation, and the accuracy achieved in the machine learning testing phase. We also compared the conditions for study reproducibility.

#### Sample Size

One of the first studies using resting-state EEG to classify individuals with depression and HCs was carried out by Ahmadlou et al [21]. Their sample was quite modest (analyzing EEG recordings of 12 patients with MDD and 12 HCs). In the same year, Puthankattil and Joseph published their research [22,23] using a slightly larger sample, with 30 patients with MDD (16 males and 14 females) and 30 controls. In 2014, Hosseinifard et al [24] and Faust et al [25] published their studies based on analyses of 90 (45 MDD+45 HC) and 60 (30 MDD+30 HC) individuals. Three studies published in 2015 had similar samples: Acharya et al [26] with 30 (15 MDD+15 HC) and Bairy et al [27] with 60 (30 MDD+30 HC, probably the same or overlapping sample as in Puthankattil and Joseph [22,23] and Faust et al [25], as it has the same group of authors and the sample description was strikingly similar), whereas Mohammadi et al [28] used a sample of 53 patients with MDD and 43 controls. A study published by Liao et al [29] also had a modest sample of 12 patients with MDD and 12 controls, whereas Mumtaz et al reported only 1 sample size used in 3 published studies with similar methodologies in 2017 [30] and 2 studies in 2018 (33 MDD+30 HC) [31,32]. Although they published their first study on spectral and fractal measures as potential markers of depression in 2013, Bachmann et al [16] added machine learning methodology to previous fractal and novel spectral (spectral asymmetry index [SASI]) analysis in their 2018 study, where their sample comprised 13 patients with MDD and 13 HCs. The study by Bachmann et al [16] is the only study that analyzed a sample consisting exclusively of female participants (which makes sense given that women present a 50% higher risk of depression). Our study [33,34] from the same year described the results of an analysis carried out on a sample of 21 patients with MDD and 20 controls. Overall, there were 362 patients with MDD and 340 age-matched HCs.

The differences between samples vary in terms of the tests used to confirm the status of the patients with MDD (DSM-IV, International Classification of Diseases, Beck Depression Scale, and Montgomery-Asberg Scale) as well as the state when recording (open or closed eyes or both). Studies also differed in terms of medication status of patients with MDD, with some being all unmedicated participants [24] with defined medication washout at 6 weeks, others being medication naïve, others stating that their patients were medicated [33], and others that did not even report on the medication status [27].

#### Method of Recording the EEG

Another important aspect when comparing the selected studies was how the researchers recorded the resting-state EEG, under what conditions, and using how many electrodes (concerning the standard used in the *Methods* section). Ahmadlou et al [21] analyzed a 3-min resting-state EEG, with closed eyes, from only frontal positions (using 7 out of 19 electrodes in the 10/20 standard; namely, Fp1, Fp2, Fz, F3, F4, F7, and F8), as they focused on previous findings on stable frontal asymmetry in depression. They separately analyzed the left and the right hemisphere positions and used a sampling rate of 256 Hz. Puthankattil and Joseph [22,23], Faust et al [25], and Acharya et al [26] analyzed only 4-position EEG recordings, 2 on the left hemisphere and 2 on the right hemisphere: FP1-T3 and FP2-T4; Bairy et al [27] did not report on the positions used for analyses in the *Methods* section, simply stating that only positions from the left side of the brain were taken into account. Puthankattil and Joseph [22] reported that the recording lasted for 5 min, but information on eye condition was not included; they (similar to Faust et al [25] and Acharya et al [26]) used a sampling frequency of 256 Hz. Alternately, Hosseinifard et al [24] analyzed recordings from all 19 electrodes (10/20 standard), with a sampling frequency of 1 kHz. Liao et al [29] analyzed recordings from 30 electrodes recorded for 5 min. In our research, we also analyzed all the positions on the cap and used a 1 kHz sampling rate. It is important to stress here that Bachmann et al [35], in their work from 2013, stated that they found that physiological complexity is elevated on all electrodes; therefore, they decided to use only 2 electrodes for further analysis (and in their 2018 work [16], they focused on detection of the EEG signal from just 1 electrode). In comparison with previously mentioned studies that also used resting-state EEG, we confirmed that the number of electrodes is important, as principal component analysis (PCA) readings reveal that each electrode offers its own contribution to the results [34].

#### Subbands Versus Broadband

Ahmadlou et al [21] used wavelets to decompose the raw EEG signal into 5 standard subbands (gamma, beta, alpha, theta, and delta), but they also analyzed the broadband signal. Furthermore, they attempted to separately analyze the left- and right-side brain recordings but confirmed that previously significant differences disappeared when the left-right series were combined. Bachmann et al [16,35] used their own previously tested novel spectral index (SASI; based on standard subbands), and Hosseinifard et al [24] also used standard subbands. Puthankattil and Joseph [22,23] used wavelet for signal decomposition, as did Faust et al [25], whereas Acharya et al [26] (like Čukić et al [33]) used the broadband signal for analysis. Bairy et al [27] used cosine transform to decompose the signal before further analysis, although wavelets and cosine transform are also considered to be reductionist approaches, as is the Fourier approach [12]. It is not clear whether all the researchers are aware that until now no physiological significance of standard subbands of EEG use is confirmed, but their use in electrophysiology is so deeply rooted that it remains inevitable [12].

#### Preprocessing

Of the numerous potential options for preprocessing the signal, some common practices may be found in all the papers that were reviewed. For example, artifact removal may be performed either automatically or manually [24]. As EEG signals are always nonstationary, nonlinear, and noisy, researchers usually determine the trade-off in every aspect of the stages of analysis. For example, if the artifacts are manually removed, another type of artifact is immediately introduced into the signal. In addition, if independent component analysis (ICA) or other artifact removal techniques are used, the intrinsic signal dynamics could be changed. As stated by Goldberger et al [17], physiological signals are the richest in information when minimally preprocessed. Another option is to observe every trace and locate where the artifacts are present and subsequently select epochs for analysis from artifact-free sections (minimally changing the signal under study, as in the study by Čukić et al [33]).

We are aware that a mathematical connection exists between the Fourier transform and, for example, the Higuchi fractal dimension [11]; thus, it is clear that if one wants to calculate the fractal dimension on time series, the use of Fourier before this calculation is redundant. ICA also affects the electrophysiological signal if used for artifact removal. Perhaps those who are applying several methods of preprocessing of electrophysiological signals are unaware of some details of the consequences of signal processing.

#### Feature Extraction and Feature Selection

Feature extraction refers to the creation of features, such as calculating various fractal and nonlinear measures from chosen epochs (time series) of raw signal traces. On the other hand, feature selection (or reduction of the problem dimensionality) helps to remove those features that are redundant or irrelevant. In this group of publications, different authors used different combinations of the two: Ahmadlou et al [21] used 2 different algorithms for fractal dimension calculations, whereas Higuchi and Katz used features and later attempted to compare them in terms of final accuracy. After averaging both the Higuchi fractal dimension (HFD) and the Katz fractal dimension (KFD) values, they applied the analysis of variance to assess the ability of a feature to discriminate between groups based on variations both between and within groups. Puthankattil and Joseph [22] used wavelet entropy as a feature (8-level multiresolution decomposition method of discrete wavelet transform [DWT] was used), and relative wavelet energy (RWE) analysis provided information about the signal energy distribution at different decomposition levels; 12 features were extracted for training and testing. An additional 9 features included RWE values for different frequency bands and 2 were obtained by observing the trend of the variation of the average RWE of EEG signals (RWE is higher in depression). Hosseinifard et al [24] used spectral power together with HFD, correlation dimension, and the largest Lyapunov exponent (LLE) as EEG features. Faust et al [25] used wavelet packet decomposition (WPD; Db8 wavelet) to extract appropriate subbands from the raw signal.

The results of our cross-section analysis are summarized in chronological order in Table 1.

The extracted subbands were input to calculate several entropy measures: bispectral entropy (Ph, including higher order spectra technique, from Fourier analysis), Renyi entropy, approximate entropy, and sample entropy (SampEn).

The extraction of the subband process consisted of sending the original data through a sequence of down-sampling and low-pass filters that defined the transfer function (similar to classical spectra analysis, which distorts the information content of the signal, according to Klonowski [12]). In addition, before that extraction, researchers claim that high-frequency components did not contribute relevant information (contrary to our findings [33,34]), and they were also removed. After using the Student *t* test to evaluate features, several classification algorithms were applied. Acharya et al [26] applied 15 different spectral and nonlinear measures for feature extraction: fractal dimension (HFD), LLE, SampEn, detrended fluctuation analysis (DFA), Hurst exponent, higher order spectra features (weighted center of bispectrum, W_Bx and W_By), bispectrum phase entropy, normalized bispectral entropy and normalized bispectral squared entropies (Ent2 and Ent3), and recurrence quantification analysis parameters (determinism, entropy, laminarity [LAM], and recurrent times). These extracted features are ranked by the *t* value. There is no information as to whether they were calculated with standard EEG subbands or broadband signals (similar to the classical spectral measure or high-order spectra using Fourier analysis, they must have been computed in subbands, but this was not mentioned). After numerous trials, the authors, based on a comparison of values to formulate the Depression Diagnosis Index, decided to only consider LAM, W_By, and SampEn, without proper justification. They declared that “DDI is a unique formula that yields non-overlapping ranges for normal and depression classes.” This (probably) heuristically obtained index is used here instead of the more commonly utilized classifiers [26]. Mohammadi et al [28] applied linear discriminant analysis (LDA) to map features into a new feature space (data evaluation phase) and genetic algorithm (GA) to identify the most significant features. Hosseinifard et al [24] used the leave-one-out cross-validation (LOOCV) method for the training data set. A GA was used for feature selection. The population size was established at 50 and cross-over at 80% (they also attempted PCA, but the GA significantly outperformed it). Bachmann et al [16] used the SASI spectral measure but also calculated the HFD, DFA, and Lempel-Ziv complexity (LZC) as features. In our research, we combined 2 nonlinear measures as features extracted from the signal (HFD and SampEn). Later, we decorrelated them and used PCA to reduce the dimensionality of the problem [33]. Bairy et al [27] calculated the SampEn, correlation dimension, fractal dimension, Lyapunov exponent, Hurst exponent, and DFA on DWT coefficients, and the characteristic features were ranked by *t* value. Liao et al [29] proposed a method based on scalp EEG and robust spectral spatial EEG feature extraction based on the kernel eigen-filter-bank common spatial pattern (KEFB-CSP). They first filter the multichannel EEG signals (30 electrode traces) of each subband from the original sensor space to a new space where the new signals (ie, CSPs) are optimal for the classification between patients with MDD and HCs. Finally, they implement kernel PCA to transform the vector containing the CSPs from all frequency subbands to a lower-dimensional feature vector called KEFB-CSP.

**Table 1:** A comparison of the previously mentioned studies comparing several characteristics, including their accuracy on the classification task.

Study	Sample (MDD^a^+HC^b^)	Electrodes, frequency (Hz)	Preprocessing	Features	ML^c^ models	Accuracy (%)
Ahmadlou et al, 2012 [21]	12+12	7, 256	Wavelets and spectral bands (Fourier), bootstrap	Higuchi and Katz FD^d^	Enhanced probabilistic neural networks	91.30
Puthankattil and Joseph, 2012 [22]	30 (16 M^e^+14 F^f^)+30	4, 256	Wavelet, total variation filtering, multiresolution decomposition	Wavelet entropy	RWE^g^, artificial feed forward networks	98.11
Hosseinifard et al, 2014 [24]	45+45	19, 1 kHz	Standard spectral bands	Power, DFA^h^, Higuchi, correlation dimension, Lyapunov exponent	KNN^i^, LR^j^, linear discriminant	90
Faust et al, 2014 [25]	30+30	4 (2 left, 2 right), 256	Wavelet package decomposition	ApEn^k^, SampEn^l^, REN^m^, bispectral phase entropy	PNN^n^, SVM^o^, DT^p^, KNN, NB^q^, GMM^r^, Fuzzy Gueno Classifier	99.50
Bairy et al, 2015 [27]	30+30 (left brain only)	N/A^s^	Discrete cosine transform	SampEn, FD, CD^t^, Hurst exp, LLE^u^, DFA	DT, KNN, NB, SVM	93.80
Acharya et al, 2015 [26]	15+15	2 left, 2 right, 256	Broadband	FD, LLE, SampEn, DFA, H^v^, W-Bx^w^, W_By^x^, EntPh^y^, Ent1^z^, DET ^aa^, ENTR^ab^, LAM^ac^, T2 (DDI)^ad^	SVM, KNN, NB, PNN, DT	98
Mohammadi et al, 2015 [28]	53+43	28 (10/10), 500	Standard bands/FFT^ae^, LDA^af^, genetic algorithm	Spectral only	DT	80
Puthankattil and Joseph, 2014 [23]	30+30	4, 256	Wavelet package decomposition	Wavelet entropy, approximate entropy	NN^ag^	98
Liao et al, 2017 [29]	12+12	30, 500	Common spatial pattern	Spectral (common spatial pattern)	KEFB-CSP^ah^	80
Mumtaz et al, 2018 [30]	34/18 F+30/9 F^ai^	19, 256	REST^aj^	Synchronization likelihood	SVM, LR, NB	87.50
Mumtaz et al, 2017 [31]	33+30	19 (EO^ak^, EC^al^), 256	Fourier	Alpha interhemispheric asymmetry	LR, SVM, NB	98.40
Mumtaz et al, 2018 [32]	34+30	19, 256	10-fold cross-validation	Power, asymmetry, wavelet coefficients, Z-score	LR	94
Bachmann et al, 2018 [35]	13+13	1, 1 kHz	Fourier	HFD^am^, DFA, Lempel-Ziv complexity, and SASI^an^	Logistic regression	88
Čukić et al, 2018/2020 [33,34]	26+20	19, 1 kHz	Broadband EEG^ao^, 10-fold cross-validation, PCA^ap^	HFD+SampEn	MP^aq^, LR, SVM (with linear and polynomial kernel), DT, RF^ar^, NB	97.50

^a^MDD: major depressive disorder.

^b^HC: healthy control.

^c^ML: machine learning.

^d^FD: fractal dimension.

^e^M: male.

^f^F: female.

^g^RWE: relative wavelet energy.

^h^DFA: detrended fluctuation analysis.

^i^KNN: K-nearest neighbor.

^j^LR: linear regression.

^k^ApEn: approximate entropy.

^l^SampEn: sample entropy.

^m^REN: Renyi entropy.

^n^PNN: probabilistic neural network.

^o^SVM: support vector machine.

^p^DT: decision tree.

^q^NB: naïve Bayes.

^r^GMM: Gaussian mixture model.

^s^N/A: not applicable.

^t^CD: correlation dimension.

^u^LLE: largest Lyapunov exponent.

^v^H: Hurst exponent.

^w^W-Bx: higher order spectra features (weighted center of bispectrum [W_Bx]; Acharya et al [26]).

^x^W_By: higher order spectra features (weighted center of bispectrum [W_By]; Acharya et al [26]).

^y^EntPh: bispectrum phase entropy.

^z^Ent1: normalized bispectral entropy.

^aa^DET: determinism.

^ab^ENTR: entropy.

^ac^LAM: laminarity.

^ad^T2 (DDI): recurrent times.

^ae^FFT: fast Fourier transform.

^af^LDA: linear discriminant analysis.

^ag^NN: neural network.

^ah^KEFB-CSP: kernel eigen-filter-bank common spatial pattern.

^ai^34 depression patients (among them 18 females) and 30 healthy controls (of those 9 were female).

^aj^REST: reference electrode standardization technique.

^ak^EO: eyes opened.

^al^EC: eyes closed.

^am^HFD: Higuchi fractal dimension.

^an^SASI: spectral asymmetry index.

^ao^EEG: electroencephalogram.

^ap^PCA: principal component analysis.

^aq^MP: multilayer perceptron.

^ar^RF: random forest.

#### Classifiers Used and Validation

Ahmadlou et al [21] used averaged, calculated KFD and HFD values (dividing it between the left and right electrodes and averaging it) as features for enhanced probabilistic neural networks. Puthankattil and Joseph [22] used the RWE and artificial feedforward neural network, and Hosseinifard et al [24] used K-nearest neighbor (KNN), LDA, and linear regression (LR) classifiers. Two-thirds of the sample was used for the training phase and the remainder was used for the test set. Faust et al [25] used WPD (Db8 wavelet) to extract appropriate subbands from the raw signal. The extracted subbands were input for calculating the entropy measures. They used a Gaussian mixture model, decision trees (DTs), KNN, naïve Bayes classifier (NBC), probabilistic neural networks, fuzzy Sugeno classifier, and support vector machine (SVM) and 10-fold cross-validation. Acharya et al [26] used SVM with a polynomial kernel of order 3, but the validation method was not reported. Mohammadi et al [28] built predictive models using a DT. The classifiers used in the research by Bairy et al [27] were DT, SVM, KNN, and naïve Bayes (NB). SVM employs a radial basis function. The model applied in the study by Mohammadi et al [28] revealed an average accuracy of 80% (MDD vs HC). There is no clear information regarding the verification of reliability of their high accuracy or internal and external validation (in terms of good generalization). Bachmann et al [16] used features for classification via logistic regression with LOOCV. As evident that characterization of the resting-state EEG with nonlinear measures leads to very accurate classification, we applied the 7 most popular classifiers in our research: multilayer perceptron, LR, SVM with a linear and polynomial kernel, DT, random forest, and NBC, discriminating EEG between HC subjects and patients diagnosed with depression [33]; 10-fold cross-validation was used in that work.

#### Classification Accuracy

Ahmadlou et al [21] found that MDD and non-MDD are more separable in the beta band based on HFD (contrary to previous belief that differentiation is best in the alpha band) and that HFD in both beta and gamma bands is higher in patients with MDD than in healthy participants. This implied a higher complexity of signal recorded from the frontal cortices (according to their data, the left frontal lobe is more affected). On the basis of HFD (which performed better than KFD), they obtained a high accuracy of 91.3%. Puthankattil and Joseph [22] obtained artificial neural network performance with an accuracy of 98.11% (normal and depression signals). Sensitivity was 98.7%, selectivity was 97.5%, and specificity was 97.5%. In the study by Hosseinifard et al [24], classification accuracy was the highest in the alpha band for LDA and LR, both of which reached 73.3% (the worst was KNN in the delta and beta bands and LDA in the delta band with 66.6%). The highest accuracy in the experiment was obtained using the LR and LDA classifiers. The accuracy of all classifiers increased when the signal was characterized by nonlinear features, not classical power (LR reached 90% with the correlation dimension). The conclusion was that “nonlinear features give much better results in the classification of depressed patients and normal subjects,” as opposed to the classical features. It was also concluded that patients with depression and controls differ more in the alpha band than in the other bands, especially in the left hemisphere [24]. Faust et al [25] applied 10-fold stratified cross-validation. The accuracy was 99.5%, sensitivity was 99.2%, and specificity was 99.7%. Contrary to Hosseinifard et al [24], they claim that the EEG signals from the right part of the brain are better at discriminating individuals with depression. In the study by Acharya et al [26], features are ranked by the *t* value and are fed to classifiers one by one, obtaining an accuracy of >98%, sensitivity of >97%, and specificity of >98.5%. This best result is reportedly obtained through the use of SVM with a polynomial kernel of order 3 (for both left and right hemispheres; they used averaged values for the left and right hemispheres), although SVM was discarded in previous papers by the same authors. The text is ambiguous when stating “features are fed to SVM classifier” and the following sentence “SVM classifier yielded the highest classification performance with the average accuracy...” [26]. In the study by Faust et al [25], the accuracy was 99.5%, sensitivity was 99.2%, and specificity was 99.7%. Unlike Hosseinifard et al [24], they claim that EEG signals from the right part of the brain better discriminate individuals with depression. Bairy et al [27] reported an accuracy of 93.8%, sensitivity of 92%, and specificity of 95.9%. It is impossible to determine whether internal or external validation was performed or determine the details, for example, the method used to calculate the fractal dimension (that description was not reported, limiting comparisons with our work on HFD). Thus, this study, which claims such high accuracy, has limited reproducibility. Liao et al [29] achieved 80% accuracy using KEFB-CSP, and Mumtaz et al [30] reported an SVM classification accuracy of 98%, LR classification accuracy of 91.7%, and NB classification accuracy of 93.6%. Bachmann et al [35] reached a maximum accuracy of 85% with HFD and DFA and also with HFD and LZC and, for only 1 nonlinear measure, a maximum accuracy of 77%. The average accuracy among classifiers reported by Čukić et al [33] ranged from 90.24% to 97.56%. Among the 2 measures, SampEn demonstrated better performance. When compared with the previously mentioned studies that also used resting-state EEG, it was possible to confirm that the number of electrodes is an important factor, as PCA readings demonstrate that every electrode offers its own contribution to the results [33,34].

In conclusion, we cannot state that all the mentioned studies provide sufficient information for replication, as it is clearly not the case with Bairy et al [27], who did not mention the method used to calculate the fractal dimension and the algorithm. Others concentrated on classification improvement but did not implement all the measures necessary to reach unwarranted optimism in their results. Finally, all the studies (including ours, although as declared, it was a pilot study) had very modest sample sizes, affecting the model’s generalizability. A summary of comparisons of analysis of signals in the literature has been illustrated in Table 2, and a summary of comparisons with regard to the classifications applied is provided in Table 3.

**Table 2:** Summary of the abovementioned comparisons of analysis of signals in the literature.

Analysis of signal	Number of electrodes	Subbands	Filtering	Method of analysis	Feature extraction
Common	1, 3, or 7 (prefrontal)	Standard subbands	Preprocessing on site	Fourier and its derivatives	*t* test or ANOVA^a^
Recommended	19+ (all electrodes)	Broadband	Minimal preprocessing	Fractal and nonlinear	PCA^b^ or GA^c^

^a^ANOVA: analysis of variance.

^b^PCA: principal component analysis.

^c^GA: genetic algorithm.

**Table 3:** Summary of the abovementioned comparisons with regard to the classifications applied.

Classification	Sample size	Data collection	Feature selection	Validation	Model	Accuracy
Common	12-40	1 site	Spectral analysis	Often missing	SVM^a^	Typically >95% or 99%
Recommended	>50-100	Multiple sites/collaborative (possible extraction from MRI^b^ sets)	Nonlinear analysis	Internal plus external validation on unseen data	LASO^c^, embedded regularization	ROC^d^ curve application/more realistic results

^a^SVM: support vector machine.

^b^MRI: magnetic resonance imaging.

^c^LASO: the name of the algorithm; a type of linear regression that uses shrinkage.

^d^ROC: receiver operating characteristic.

### Interventional EEG Studies

Studies have also been published during the same time interval (2008-2019) based on EEG registration, but unlike the previously mentioned work, they opted to use a stimulus (so, not resting-state EEG), a sound stimulation, or evoked response potentials (ERPs). Therefore, we briefly discuss their results. Kalatzis et al [36] published the first study on the SVM-based classification system to discriminate depression using the P600 component of ERP signals. EEG was recorded on 15 electrodes, and the sample consisted of 25 patients and an equal number of HCs. The outcomes of SVM classification were selected by the majority vote engine. Classification accuracy was reportedly 94% when using all leads and between 92% and 80% when using only the right or left electrode positions for classification. They concluded that their findings support the hypothesis that depression is associated with the dysfunction of right hemisphere mechanisms mediating the processing of information that assigns a specific response to a particular stimulus. Lee et al [37] attempted to predict the treatment response of MDD. Their study was designed to verify whether the connectivity strength of resting-state EEG could be a potential biomarker (ROC curve was 0.6 to 0.8) used to answer this question. They concluded that “...the stronger the connectivity strength, the poorer the treatment response.” The experiment also suggested that frontotemporal connectivity strength could be a potential biomarker to differentiate between responders and slow responders or nonresponders in MDD. We attempted to compare our results, but their sampling frequency was as low as 100 Hz, making this comparison difficult. In a 2011 study, Cavanagh et al [38] analyzed EEG recordings from 21 medication-free patients with MDD and 24 HCs when performing a probabilistic reinforcement learning task. They measured the EEG response to error feedback, which may demonstrate selective alteration of avoidance learning, which is important in MDD. Khodayari-Rostamabad et al [39,40] probed machine learning methodology as a prediction model for a successful outcome of SSRI medication in MDD based on resting-state EEG recorded before the treatment. The sample consisted of 22 participants (11 males and 11 females). For the experiment, only 16 electrodes were used (10/20 standard) in open and closed eyes, recording for 6.5 min and combining sections into 6 files per person. The Welch model analysis yielded various spectral measures but mentioned *only as candidate features* because they did not wish to state which feature would have predictive power in advance. After selecting the features extracted from the EEG, the authors included them in the factor analysis model, whose output is the predicted response in the form of a likelihood value; the leave-one-out randomized permutation cross-validation procedure was used for validation. For visualization (and reduction of dimensionality), they used kernelized PCA. The authors did not perform assessment on unseen samples, and they did not compare the features with HCs, relying solely on spectral measures of their modest sample. They reported an overall prediction accuracy of 87.9%.

A study from 2014 attempted to predict the depression treatment response [41]. The authors claimed that no difference exists between MDD and HC in nonlinear EEG measures (using LZC), but they somehow came to the conclusion that nonlinear measures add value to their research. They claim that theirs is the first study to use nonlinear metrics to predict the outcome of depression treatment (repetitive transcranial magnetic stimulation [rTMS] in their case). According to their reported method, the potential cause could be the focus on only one specific band and not on the analysis of broadband signals. Many subsequent researchers (and previous ones) managed to find significant differences through the use of nonlinear measures for this type of detection task [16,21,24,28,30,33]. They also claimed that they were *the first* to use complexity measures in this task. Nandrino and Pezard [41] used this approach in the analysis of EEG in depression in 1994, as did several other research groups. Bachmann et al [16] applied the same methodology (LZC) and demonstrated significant differentiation between patients and controls. Mumtaz et al [30,31] used spectral measures in several papers but found a useful difference in predicting treatment outcome in depression.

Similar to Shahaf et al [42], Etkin et al [43] applied machine learning in the task of predicting medication therapy outcomes in MDD through cognitive testing. They used pattern classification with cross-validation to determine individual patient-level composite predictive biomarkers of antidepressant outcome based on test performance and obtained 91% accuracy.

Erguzel et al [44] tested their optimized classification methods on 147 participants with MDD treated with rTMS. They tested the performance of a GA and a back-propagation neural network; they were evaluated using 6-channel pre-rTMS EEG patterns of theta and delta frequency bands. Using the reduced feature set, they obtained an increase of 0.904 in the ROC curve (area under the curve). Zhang et al [45] explored neural complexity in patients with poststroke depression in a resting-state EEG study. Their sample consisted of 21 poststroke patients with depression (PSD), 22 patients with ischemic stroke but no depression (PSND), and 15 HCs. A total of 16 electrodes were used for recording resting-state EEG. LZC was used to assess changes in complexity from EEG. PSD (depressed) presented lower neural complexity compared with PSND (nondepressed) and control subjects for the entire brain region. LZC parameters used for the recognition of PSD possessed >85% specificity, sensitivity, and accuracy, suggesting the feasibility of LZC as a screening indicator for PSD. In addition, there were 2 antidepressive treatment nonresponse prediction studies by Shahaf et al [45] and Al-Kaysi et al [46]. Shahaf et al [45] developed a new electrophysiological attention-associated marker from a single channel (2 electrodes: Fpz and 1 earlobe) using 1-min samples with auditory oddball stimuli that was capable of detecting treatment-resistant depression (26 patients and 10 controls). Al-Kaysi et al [46] aimed to predict the transcranial direct current stimulation (tDCS) treatment outcome of patients with MDD using automated EEG classification. They accurately predicted 8 out of 10 participants when using FC4-F8 (with an accuracy of 76%) and 10 out of 10 participants when using CPz-CP2 (92% accuracy). This finding demonstrates the feasibility of using machine learning to identify patients responsive to the tDCS treatment. Cai et al [47] used only 3 electrodes on prefrontal positions to record the signal when stimulating their participants with a sound. They claim that owing to the small number of electrodes that can be easily positioned, their method has excellent potential for use in clinics. They used an electrophysiological database consisting of 92 patients with depression and 121 HCs; resting-state EEG was recorded using sound stimulation (pervasive prefrontal lobe electrodes were used on the Fp1, Fp2, and Fpz positions). After denoising (finite impulse response filter), they combined the Kalman derivative formula and the discrete wavelet transformation and an adaptive predictor filter; a total of 270 linear and nonlinear features were extracted (it is not clear what they were). Feature selection was minimal-redundancy-maximal-relevance, which reduced the dimensionality of the feature space. Four classification methods were applied: SVM, KNN, DTs, and artificial neural networks. For evaluation, they used 10-fold cross-validation. KNN presented the highest accuracy at 79.27%. Jaworska et al [48,49] published 2 papers. In the first study [48], they examined a variation of pretreatment EEG to predict depression treatment success, and in the second work [49], they performed a 12-week machine learning study to predict the outcome of pharmacology treatments in 51 patients with MDD. They used both electrophysiological and demographic data (including the Montgomery-Asberg Depression Rating Scale scores before and after treatment) as well as source-localized current density and random forest for classification, with 78% to 88% accuracy depending on model complexity. They also used kernel PCA to reduce and map important features. Similar to the abovementioned research, this lays the groundwork for studies on personalized, *biomarker*-based treatment approaches. For this group of studies, it is clear that methodology comparisons are challenging, but they are part of the same effort to show that not only detection but also monitoring and predicting the pace of recovery, or output of the treatment (sometimes called *responders* detection), is possible. The problem with both detection and interventional studies tends to be the modest sample sizes and almost complete absence of an external validation process (on previously unseen data, from an independent sample), which puts their high reported accuracies into question.

## Discussion

Most of the publications included in our review presented high accuracy in classifying individuals with depression and healthy participants based on their resting-state EEG, although they utilized various combinations of features and machine learning models. Although direct comparison is challenging, the common denominator for all presented studies can be summarized as a comparison of the methodological steps that are inevitable in this kind of research, in which certain features, previously found to be characteristic for depression, were used to feed classifiers of their choosing.

Several approaches may be used to examine the changes in the complexity of the EEG characteristic of depression. Researchers have reached a consensus that depression is characterized by high EEG complexity, compared with healthy peers [13]. Changes in functional connectivity characteristic of depression are demonstrated in the current literature, whether using fMRI, fractional anisotropy [50,51], or graph theory analysis based on EEG signals [52]. It is possible that decreased functional connectivity may be reflected by increased excitability of the cortex; thus, a difference in EEG between people diagnosed with depression and HCs could be detected [16,21,24,25]. The most important conclusion of the review by de la Torre-Luque and Bornas [13] was that “EEG dynamics for depressive patients appear more random than the dynamics of healthy non-depressed individuals.” It is also accepted that the use of more than one nonlinear measure should be standard as different measures detect unique features of the EEG signals, “revealing information which other measures were unable to detect” [52,53].

The classification of patients diagnosed with depression and HCs can be considered as a first step in exploring the potential for prediction. Differentiation between episode and remission is also possible [54]. The prediction of clinical outcomes or relapses (eg, after incomplete remission or relapse in recurrent depression) would be of great clinical significance. However, there are several challenges, both methodological and statistical, to the development of a model to predict a specific clinical outcome for previously unseen individuals. A group of authors elucidated some of the risks, pitfalls, and recommended techniques to improve model reliability and validity in future research [4,7,55-58]. The authors declared that neuroimaging researchers who begin to develop such predictive models are typically unaware of some of the required considerations to accurately assess model performance and avoid inflated predictions (so-called *unwarranted optimism*) [4,55,56,59]. The common characteristics of this type of research are as follows: classification accuracy is typically 80% to 90% overall, the sample size tends to be small to modest, and samples are usually gathered from a single site. SVM and its variants are very popular, but the use of embedded regularization frameworks is recommended, at least with the absolute shrinkage and selection operator [7]. LOOCV and k-fold cross-validation are also popular procedures for validation (for model evaluation), and model generalization capability is typically untested on independent samples [7]. The rarely employed Vapnik-Chevronenkis dimension [60] should be of standard use for model evaluation or reduction. A lack of external validation is common in most current studies. From a methodological point of view, those problems must be resolved. For example, the problem of generalization. Generalization is the ability of a model that was trained in one data set to predict patterns in another unseen data set. When testing generalizability, we are examining whether a classification is effective in an independent (not shown to the previous algorithm) population. When developing a model, one does not wish to train the classifier on a general sample characteristic; for example, if using nonlinear measures, they may differ because some measures change with age [17] or may be characteristic of a certain gender [61,62]. Some authors refer to these as *nuisance variables* because the algorithm learns to recognize that particular data set with all of its characteristics. Overfitting is common and consequently the treatment of nuisance variables. Overfitting takes place when “a developed model perfectly describes the overall aspects of the training data (including all underlying relationships and associated noise), resulting in fitting error to asymptotically become zero” [7]. Thus, the model will be unable to predict what we want on unseen (test) data. The sample size is usually small to modest (typically <100; in the study by Chekroud et al [56], for example, it is >4000 because of the use of a collaborative data set). Hence, balancing the model’s complexity against the sample size is essential for improving the prediction accuracy for unseen (test) data [7]. How can this goal be achieved? By collecting more data. The collection of other more expensive neuroimaging data would be a potential solution to establish a standard set-up and start collaborative projects, as a single site is usually not sufficient to ensure a large sample size. In EEG collection, a desired model could be one that is made up of large collaborative projects such as RDoC, STAR*D, and IMAGEN. In addition, corecording with fMRI and magneto encephalography may be a solution [57]. Another option could be the use of wireless EEG caps. Although present wireless EEG caps are still quite expensive (Epoch, ENOBIO Neuroelectrics, and iMotions, to mention just a few), they can be used for research in the environment without restraining the patient or even to monitor individuals recovering from severe episodes. If wireless EEG recorders would become accessible soon, early detection and timely intervention will most likely prevail rapidly. In frameworks such as the National Institute of Mental Health Research Domain Criteria and European Roadmap for Mental Health Research, which aim to discover stratifications based on biological markers that cut across current classifications [58], this should be possible. Through large collaborative efforts, the conditions may be met to extract genuinely reliable models for clearly defined neuromarkers for future clinical use [57]. Large-scale imaging campaigns and the collection of general population data are essential conditions for the transfer of these research findings to clinics. By permitting regular medical checkup data to become a part of such organized collaborative efforts, patients would also contribute to the improvement of this precise diagnostic in the near future. According to Kraiij [63], the 4P concept for health care improvement stands for prediction, prevention, personalization, and participation. It has been suggested that health care focuses too much on disease treatment and not enough on its prevention. It has also been observed [58] that treatment and diagnosis tend to be based on population averages. In some cases, treatment has negative effects. Therefore, there is much room for improvement (and for the other 3 Ps aside, personalization). Data collection, analysis, and sharing play an important role in the improvement of health care. The first project to implement the 4Ps is the SWELL (Smart Reasoning for Well-being at Home and at Work) project, part of the Dutch national ICT program, COMMIT (between 2011 and 2016 in the Netherlands, Leiden University). There is also an option of testimonial data sharing that is already official, eg, in Austria.

Whelan and Garavan [55] addressed overfitting and many other methodological issues. They revealed how regression models may incorrectly appear to be predictive. They also described methods for quantifying and improving model reliability and validity. The authors conclude that “...perhaps counterintuitively to those who deal primarily with a general linear model, optimism increases as a function of the decreasing number of participants and the increasing number of predictor variables in the model (the model appears better as sample size decreases)” [55].

Although it has been shown that small sample sizes and a lack of external validation lead to unwarranted optimism, most published research does not embrace these principles as standard practice [64]. Collecting additional data may resolve this issue. The theory of data mining is clear; all models work best on larger samples. The repository may be used to test a developed model on an unseen cohort. We learned that statistics needs “to stop making fools of ourselves” [65]. Data mining is the art of finding meaning from supposedly meaningless data. Peduzzi et al [66] showed the optimal number of events per variable in logistic regression analysis.

A minimum rate of 10 cases per predictor is common [64], although it is not a universal recommendation [67]. Optimism may also be avoided with the introduction of the regularization term [68]. In addition, using previous information to constrain model complexity relying on Bayesian approaches is recommended. Bootstrapping [69] is another useful method, as is cross-validation [59]. Cross-validation tests the model’s ability to generalize and involves separating the data into subsets. Both Kohavi [70] and Ng [64] described this technique. In addition, an effective and efficient 10-fold cross-validation, Elastic Net, is useful for optimizing parameters. Ng [64] stated that “...optimism becomes unreliable as the probability of overfitting to the test data increases with multiple comparisons.” One can use several functions available in MATLAB (MathWorks) such as lassoglm, bootstrap for bootstrap sampling, or several functions for Bayesian analysis or the function *crossvalind* for testing sets and cross-validation.

In conclusion, when discussing the importance of maintaining completely separate training and test subsets, Whelan and Garavan [55] stated the following: “any cross-contamination will result in optimism.” We could not agree more. Additional research is necessary to reframe nosology in psychiatry and to help support the patient’s journey to remission. We hope that many people will benefit from the cloud-based services provided by already digitized health care institutions.

## Abbreviations

DFA: detrended fluctuation analysis
DSM-IV: Diagnostic and Statistical Manual of Mental Disorders, fourth edition
DT: decision tree
DWT: discrete wavelet transform
EEG: electroencephalogram
ERP: evoked response potential
fMRI: functional magnetic resonance imaging
GA: genetic algorithm
HC: healthy control
HFD: Higuchi fractal dimension
ICA: independent component analysis
KEFB-CSP: kernel eigen-filter-bank common spatial pattern
KFD: Katz fractal dimension
KNN: K-nearest neighbors
LAM: laminarity
LDA: linear discriminant analysis
LLE: largest Lyapunov exponent
LOOCV: leave-one-out cross-validation
LR: linear regression
LZC: Lempel-Ziv complexity
MDD: major depressive disorder
MRI: magnetic resonance imaging
NB: naïve Bayes
NBC: naïve Bayes classifier
PCA: principal component analysis
PSD: poststroke patients with depression
PSND: patients with ischemic stroke but no depression
ROC: receiver operating characteristic
rTMS: repetitive transcranial magnetic stimulation
RWE: relative wavelet energy
SampEn: sample entropy
SASI: spectral asymmetry index
SVM: support vector machine
tDCS: transcranial direct current stimulation
WPD: wavelet packet decomposition

## References

[1] (2017). Depression and Other Common Mental Disorders: Global Health Estimates. World Health Organization.

[2] Rush AJ, Trivedi MH, Wisniewski SR, Nierenberg AA, Stewart JW, Warden D, Niederehe G, Thase ME, Lavori PW, Lebowitz BD, McGrath PJ, Rosenbaum JF, Sackeim HA, Kupfer DJ, Luther J, Fava M (2006). Acute and longer-term outcomes in depressed outpatients requiring one or several treatment steps: a STAR*D report. Am J Psychiatry.

[3] Cipriani A, Furukawa Ta, Salanti G, Geddes Jr, Higgins Jp, Churchill R, Watanabe N, Nakagawa A, Omori Im, McGuire H, Tansella M, Barbui C (2009). Comparative efficacy and acceptability of 12 new-generation antidepressants: a multiple-treatments meta-analysis. Lancet.

[4] Gillan CM, Whelan R (2017). What big data can do for treatment in psychiatry. Curr Opin Behav Sci.

[5] Montague PR, Dolan RJ, Friston KJ, Dayan P (2012). Computational psychiatry. Trends Cogn Sci.

[6] Wang X, Krystal J (2014). Computational psychiatry. Neuron.

[7] Yahata N, Kasai K, Kawato M (2017). Computational neuroscience approach to biomarkers and treatments for mental disorders. Psychiatry Clin Neurosci.

[8] Tokuda T, Yoshimoto J, Shimizu Y, Okada G, Takamura M, Okamoto Y, Yamawaki S, Doya K (2018). Identification of depression subtypes and relevant brain regions using a data-driven approach. Sci Rep.

[9] Witten IH, Frank E, Hall MA (2005). Measuring the social interactions of people with traumatic brain injury and their communication partners: The adapted Kagan scales. DATA MINING Practical Machine Learning Tools and Techniques.

[10] Craik A, He Y, Contreras-Vidal JL (2019). Deep learning for electroencephalogram (EEG) classification tasks: a review. J Neural Eng.

[11] Kalauzi A, Bojić T, Vuckovic A (2012). Modeling the relationship between Higuchi's fractal dimension and Fourier spectra of physiological signals. Med Biol Eng Comput.

[12] Klonowski W (2007). From conformons to human brains: an informal overview of nonlinear dynamics and its applications in biomedicine. Nonlinear Biomed Phys.

[13] de la Torre-Luque A, Bornas X (2017). Complexity and irregularity in the brain oscillations of depressive patients: a systematic review. Neuropsychiatry.

[14] Nandrino J, Pezard L, Martinerie J, el Massioui F, Renault B, Jouvent R, Allilaire J, Widlöcher D (1994). Decrease of complexity in EEG as a symptom of depression. Neuroreport.

[15] Llamocca PP, Junestrand M, Cukic M, Urgeles D, Lopez V (2018). Data Source Analysis in Mood Disorder Research. XVIII Conference of the Spanish Association of Artificial Intelligence.

[16] Bachmann M, Päeske L, Kalev K, Aarma K, Lehtmets A, Ööpik P, Lass J, Hinrikus H (2018). Methods for classifying depression in single channel EEG using linear and nonlinear signal analysis. Comput Methods Programs Biomed.

[17] Goldberger AL, Peng C, Lipsitz LA (2002). What is physiologic complexity and how does it change with aging and disease?. Neurobiol Aging.

[18] Berman MG, Peltier S, Nee DE, Kross E, Deldin PJ, Jonides J (2011). Depression, rumination and the default network. Soc Cogn Affect Neurosci.

[19] Başar E, Güntekin B, Atagün I, Turp Gölbaşı B, Tülay E, Ozerdem A (2012). Brain's alpha activity is highly reduced in euthymic bipolar disorder patients. Cogn Neurodyn.

[20] Flach P (2012). Machine Learning: the Art and Science of Algorithms That Make Sense of Data.

[21] Ahmadlou M, Adeli H, Adeli A (2012). Fractality analysis of frontal brain in major depressive disorder. Int J Psychophysiol.

[22] Puthankattil SD, Joseph PK (2012). Classification of EEG signals in normal and depression conditions by ANN using RWE and signal entropy. J Mech Med Biol.

[23] Puthankattil SD, Joseph P (2014). Analysis of EEG signals using wavelet entropy and approximate entropy: a case study on depression patients. Int J Medical Heal Biomed Bioeng Pharm Eng.

[24] Hosseinifard B, Moradi MH, Rostami R (2013). Classifying depression patients and normal subjects using machine learning techniques and nonlinear features from EEG signal. Comput Methods Programs Biomed.

[25] Faust O, Ang PC, Puthankattil SD, Joseph PK (2014). Depression diagnosis support system based on EEG signal entropies. J Mech Med Biol.

[26] Acharya UR, Sudarshan VK, Adeli H, Santhosh J, Koh JE, Puthankatti SD, Adeli A (2015). A novel depression diagnosis index using nonlinear features in EEG signals. Eur Neurol.

[27] Bairy GM, Bhat S, Eugene LW, Niranjan UC, Puthankattil SD, Joseph PK (2015). Automated classification of depression electroencephalographic signals using discrete cosine transform and nonlinear dynamics. J Med Imaging Hlth Inform.

[28] Mohammadi M, Al-Azab F, Raahemi B, Richards G, Jaworska N, Smith D, de la Salle S, Blier P, Knott V (2015). Data mining EEG signals in depression for their diagnostic value. BMC Med Inform Decis Mak.

[29] Liao S, Wu C, Huang H, Cheng W, Liu Y (2017). Major depression detection from EEG signals using kernel eigen-filter-bank common spatial patterns. Sensors (Basel).

[30] Mumtaz W, Xia L, Ali SS, Yasin MA, Hussain M, Malik AS (2017). Electroencephalogram (EEG)-based computer-aided technique to diagnose major depressive disorder (MDD). Biomedical Signal Processing and Control.

[31] Mumtaz W, Xia L, Mohd Yasin MA, Azhar Ali SS, Malik AS (2017). A wavelet-based technique to predict treatment outcome for major depressive disorder. PLoS One.

[32] Mumtaz W, Ali SS, Yasin MA, Malik AS (2018). A machine learning framework involving EEG-based functional connectivity to diagnose major depressive disorder (MDD). Med Biol Eng Comput.

[33] Čukić M, Pokrajac D, Stokić M, Simić S, Radivojević V, Ljubisavljević M (2018). EEG machine learning with Higuchi’s fractal dimension and Sample Entropy as features for successful detection of depression. arXiv.

[34] Čukić M, Stokić M, Simić S, Pokrajac D (2020). The successful discrimination of depression from EEG could be attributed to proper feature extraction and not to a particular classification method. Cogn Neurodyn.

[35] Bachmann M, Lass J, Suhhova A, Hinrikus H (2013). Spectral asymmetry and Higuchi's fractal dimension measures of depression electroencephalogram. Comput Math Methods Med.

[36] Kalatzis I, Piliouras N, Ventouras E, Papageorgiou C, Rabavilas A, Cavouras D (2004). Design and implementation of an SVM-based computer classification system for discriminating depressive patients from healthy controls using the P600 component of ERP signals. Comput Methods Programs Biomed.

[37] Lee T, Wu Y, Yu YW, Chen M, Chen T (2011). The implication of functional connectivity strength in predicting treatment response of major depressive disorder: a resting EEG study. Psychiatry Res.

[38] Cavanagh JF, Bismark AJ, Frank MJ, Allen JJ (2011). Larger error signals in major depression are associated with better avoidance learning. Front Psychol.

[39] Khodayari-Rostamabad A, Hasey GM, Maccrimmon DJ, Reilly JP, de Bruin H (2010). A pilot study to determine whether machine learning methodologies using pre-treatment electroencephalography can predict the symptomatic response to clozapine therapy. Clin Neurophysiol.

[40] Khodayari-Rostamabad A, Reilly JP, Hasey GM, de Bruin H, Maccrimmon DJ (2013). A machine learning approach using EEG data to predict response to SSRI treatment for major depressive disorder. Clin Neurophysiol.

[41] Arns M, Cerquera A, Gutiérrez RM, Hasselman F, Freund JA (2014). Non-linear EEG analyses predict non-response to rTMS treatment in major depressive disorder. Clin Neurophysiol.

[42] Shahaf G, Yariv S, Bloch B, Nitzan U, Segev A, Reshef A, Bloch Y (2017). A pilot study of possible easy-to-use electrophysiological index for early detection of antidepressive treatment non-response. Front Psychiatry.

[43] Etkin A, Patenaude B, Song YJ, Usherwood T, Rekshan W, Schatzberg AF, Rush AJ, Williams LM (2015). A cognitive-emotional biomarker for predicting remission with antidepressant medications: a report from the iSPOT-D trial. Neuropsychopharmacology.

[44] Erguzel TT, Ozekes S, Tan O, Gultekin S (2015). Feature selection and classification of electroencephalographic signals: an artificial neural network and genetic algorithm based approach. Clin EEG Neurosci.

[45] Zhang Y, Wang C, Sun C, Zhang X, Wang Y, Qi H, He F, Zhao X, Wan B, Du J, Ming D (2015). Neural complexity in patients with poststroke depression: a resting EEG study. J Affect Disord.

[46] Al-Kaysi AM, Al-Ani A, Loo CK, Powell TY, Martin DM, Breakspear M, Boonstra TW (2017). Predicting tDCS treatment outcomes of patients with major depressive disorder using automated EEG classification. J Affect Disord.

[47] Cai H, Han J, Chen Y, Sha X, Wang Z, Hu B, Yang J, Feng L, Ding Z, Chen Y, Gutknecht J (2018). A pervasive approach to EEG-based depression detection. Complexity.

[48] Jaworska N, Wang H, Smith DM, Blier P, Knott V, Protzner AB (2018). Pre-treatment EEG signal variability is associated with treatment success in depression. Neuroimage Clin.

[49] Jaworska N, de la Salle S, Ibrahim M, Blier P, Knott V (2018). Leveraging machine learning approaches for predicting antidepressant treatment response using electroencephalography (EEG) and clinical data. Front Psychiatry.

[50] de Kwaasteniet B, Ruhe E, Caan M, Rive M, Olabarriaga S, Groefsema M, Heesink L, van Wingen G, Denys D (2013). Relation between structural and functional connectivity in major depressive disorder. Biol Psychiatry.

[51] Vederine F, Wessa M, Leboyer M, Houenou J (2011). A meta-analysis of whole-brain diffusion tensor imaging studies in bipolar disorder. Prog Neuropsychopharmacol Biol Psychiatry.

[52] Kim D, Bolbecker AR, Howell J, Rass O, Sporns O, Hetrick WP, Breier A, O'Donnell BF (2013). Disturbed resting state EEG synchronization in bipolar disorder: a graph-theoretic analysis. Neuroimage Clin.

[53] Burns TF, Rajan R (2015). Combining complexity measures of EEG data: multiplying measures reveal previously hidden information. F1000 Research.

[54] Čukić M, Stokić M, Radenković S, Ljubisavljević M, Simić S, Savić D (2020). Nonlinear analysis of EEG complexity in episode and remission phase of recurrent depression. Int J Methods Psychiatr Res.

[55] Whelan R, Garavan H (2014). When optimism hurts: inflated predictions in psychiatric neuroimaging. Biol Psychiatry.

[56] Chekroud AM, Zotti RJ, Shehzad Z, Gueorguieva R, Johnson MK, Trivedi MH, Cannon TD, Krystal JH, Corlett PR (2016). Cross-trial prediction of treatment outcome in depression: a machine learning approach. Lancet Psychiatry.

[57] Gillan C, Daw N (2016). Taking psychiatry research online. Neuron.

[58] Marquand AF, Wolfers T, Mennes M, Buitelaar J, Beckmann CF (2016). Beyond lumping and splitting: a review of computational approaches for stratifying psychiatric disorders. Biol Psychiatry Cogn Neurosci Neuroimaging.

[59] Efron R, Tibshirani R (2020). Improvements on Cross-Validation: The .632+ Bootstrap Method. J Am Stat Assoc.

[60] Vapnik VN (1998). Statistical Learning Theory.

[61] Ahmadi K, Ahmadlou M, Rezazade M, Azad-Marzabadi E, Sajedi F (2013). Brain activity of women is more fractal than men. Neurosci Lett.

[62] Ahmadlou M, Adeli H, Adeli A (2013). Spatiotemporal analysis of relative convergence of EEGs reveals differences between brain dynamics of depressive women and men. Clin EEG Neurosci.

[63] Kraiij W (2017). Innaugurational speech on the acceptance of his position as professor of Applied data Analytics at University Leiden. Universiteit Leiden.

[64] Ng AY (1997). Preventing overfitting of cross-validation data. Proceedings of the 14th International Conference on Machine Learning.

[65] Lakens D (2013). Calculating and reporting effect sizes to facilitate cumulative science: a practical primer for t-tests and ANOVAs. Front Psychol.

[66] Peduzzi P, Concato J, Kemper E, Holford TR, Feinstein AR (1996). A simulation study of the number of events per variable in logistic regression analysis. J Clin Epidemiol.

[67] Vittinghoff E, McCulloch CE (2007). Relaxing the rule of ten events per variable in logistic and Cox regression. Am J Epidemiol.

[68] Moons K, Donders AR, Steyerberg E, Harrell F (2004). Penalized maximum likelihood estimation to directly adjust diagnostic and prognostic prediction models for overoptimism: a clinical example. J Clin Epidemiol.

[69] Efron B, Tibshirani RJ (1993). An introduction to the Bootstrap.

[70] Kohavi R (1995). A Study of Cross-Validation and Bootstrap for Accuracy Estimation and Model Selecti. https://dl.acm.org/doi/10.5555/1643031.1643047.

